# General Behavioral Engagement and Changes in Clinical and Cognitive Outcomes of Patients with Type 2 Diabetes Using the Time2Focus Mobile App for Diabetes Education: Pilot Evaluation

**DOI:** 10.2196/17537

**Published:** 2021-01-20

**Authors:** Bryan C Batch, Susan E Spratt, Dan V Blalock, Chad Benditz, Andi Weiss, Rowena J Dolor, Alex H Cho

**Affiliations:** 1 Division of Endocrinology, Metabolism and Nutrition Duke University School of Medicine Durham, NC United States; 2 Center for Innovation to Accelerate Discovery and Practice Transformation Durham Veterans Affairs Medical Center Durham, NC United States; 3 Department of Psychiatry and Behavioral Sciences Duke University School of Medicine Durham, NC United States; 4 MicroMass Communications, Inc Cary, NC United States; 5 Division of General Internal Medicine Department of Medicine Duke University School of Medicine Durham, NC United States

**Keywords:** mobile technology, diabetes, self management support, self efficacy, illness perception

## Abstract

**Background:**

Type 2 diabetes affects 30 million Americans, representing a significant cause of morbidity and mortality. Self-management support is an important component of chronic illness care and is a key pillar of the chronic care model. Face-to-face teaching and patient education materials suffer from being static or incompatible with mobile lifestyles. Digital apps provide a self-management support alternative that is convenient and scalable.

**Objective:**

This pilot study tested the real-world deployment of a self-guided mobile app for diabetes education (Time2Focus app; MicroMass Communications Inc, Cary, NC), which utilizes evidence-based content and gamification to deliver an interactive learning experience.

**Methods:**

Primary care providers were approached for permission to invite their patients to participate. Eligible patients were 18 to 89 years of age, had a diagnosis of type 2 diabetes, hemoglobin A1c (HbA1c) ≥8% and <12% in the past 3 months, an active online patient portal account (tied to the electronic health record), and access to an iOS or Android smartphone. Interested patients were emailed a baseline survey, and once this was completed, were sent instructions for downloading the Time2Focus app. After completing all 12 levels, participants were sent a follow-up survey. The primary outcome was the change in HbA1c. Secondary outcomes included medication adherence, self-care activities, self-reporting of physical activities, diabetes self-efficacy, illness perceptions, diabetes distress scale, and users’ engagement with and rating of the app.

**Results:**

Of 1355 potentially eligible patients screened, 201 were consented. Of these 201 patients, 101 (50.2%) did not download the app. Of the 100 participants (49.8%) who downloaded the app, 16 (16.0%) completed 0 levels, 26 (26.0%) completed 1 to 4 levels, 10 (10.0%) completed 5 to 11 levels, and 48 (48.0%) completed all 12 levels of the app and the follow-up survey. Those completing one or more levels had a mean pre/post-HbA1c change of –0.41% (compared to –0.32% among those who completed zero levels); however, the unadjusted two-tailed *t* test indicated no significant difference between the two groups (*P*=.73). Diabetes self-efficacy showed a large and significant increase during app usage for completers (mean change 1.28, *P*<.001, *d*=.83). Severity of illness perceptions showed a small but significant decrease during app usage for completers (mean change –0.51, *P*=.004, *d*=.43). Diabetes distress showed a small but significant decrease during app usage for completers (mean change –0.45, *P*=.006, *d*=.41). The net promoter score was 62.5, indicating that those who completed all levels of the app rated it highly and would recommend it to others.

**Conclusions:**

Participants who engaged in all 12 levels of the Time2Focus mobile app showed an improvement in diabetes self-efficacy and a decrease in severity of illness perceptions. The decrease in HbA1c observed in app users relative to nonusers during this limited pilot study was not statistically significant. However, uptake and application of lessons learned from self-management support may be delayed. Further research is needed to address how to increase engagement through self-management support and to investigate if follow up over a longer period demonstrates a significant change in outcomes such as HbA1c.

## Introduction

Diabetes affects 30 million Americans, and is a significant cause of morbidity and mortality. The care of diabetes and complications associated with the condition contribute to extraordinary expenditures each year, with the cost of diagnosed diabetes management in the United States reaching 327 billion dollars in 2017 [1].

Diabetes self-management support (SMS) provides patients with the knowledge and skills for implementing and sustaining the coping skills and behaviors needed to self-manage their diabetes on an ongoing basis [2]. SMS is an essential component of diabetes management, considering that diabetes is a condition in which the outcomes are heavily influenced by patient behaviors such as change in diet and physical activity. Further, SMS is an important component of chronic illness care and is a key pillar of the chronic care model, which is one of the frameworks upon which the patient-centered medical home concept is based. Prior research has shown that SMS is cost-effective, and is associated with improved patient knowledge and self-care behaviors, lower hemoglobin A1c (HbA1c), lower self-reported weight, reduced hospital admissions, reduced health care costs, and reduced risk of all-cause mortality [3,4]. However, evidence shows that only 5% to 7% of people eligible for SMS ultimately receive this support [4].

The traditional model of SMS training consists of in-person individual or group sessions. However, there are numerous challenges to the consistent and ongoing provision of such support. Traditional models have the disadvantage of being inherently limited by the need for patients to be face to face with a clinician or to attend a scheduled session at a fixed time and place. In addition, providers may not be skilled in communication techniques that have been shown to be effective in influencing behavior change.

The widespread availability of mobile smartphones and digital apps offers an alternative method of providing SMS to patients. Well-designed apps can incorporate the principles of evidence-based support for health behavior change. This method has the advantage of being convenient, patient-centered, economical, and scalable.

Despite the multitude of clinical trials focused on implementation of phone apps technology for SMS training, there continues to be a gap in the uptake of SMS training and mastery of training by patients who need it most. Further, studies of effectiveness of prior apps that include educational modules in improving clinical outcomes such as HbA1c are limited. A systematic review by Adu et al [5] explored considerations when designing and implementing apps for diabetes SMS. They noted that future designs of mobile apps for SMS need to include key elements of SMS education [5].

The Time2Focus digital app for diabetes education was developed by MicroMass Communications, Inc (Cary, NC), a firm specializing in patient and health care provider behavior change. The app is intended to help patients with type 2 diabetes build practical problem-solving skills through an interactive 12-level learning experience that uses gamification and evidence-based change support principles. Examples of topics covered include healthy eating, physical activity, and diabetes self-monitoring.

Here, we present the results of a pilot study conducted at Duke Health that tested a real-world deployment of Time2Focus, a self-guided mobile app for diabetes education. Outcomes assessed include HbA1c and change in self-reported health behaviors. All activities described below were approved by the Duke University Institutional Review Board.

## Methods

### Study Population, Recruitment, and Enrollment

Enrollment began in July 2017 and was completed in March 2018. Participants were eligible if they were ≥18 years of age, had a diagnosis of type 2 diabetes, HbA1c ≥8% (and <12%) within the past 3 months, an active MyChart patient portal account, and an iOS or Android smartphone.

Participants were excluded if they had a diagnosis of type 1 diabetes, were unable to provide consent, were legally blind, could not read, or did not read and understand English. The principal investigator of the study (AC) reached out to primary care providers at Duke Primary Care, a health system–affiliated network of primary care practices in central North Carolina. The network included 26 adult continuity practices at the time that recruitment for this study began. The principal investigator requested permission from providers to contact patients who were potentially eligible for the study. Participants were also identified via endocrinology clinic–based provider outreach. Recruitment letters were sent to these patients and signed by their primary care providers. The letters briefly described the study and allowed patients to opt out of being contacted. After informed consent was obtained, participants were contacted by MicroMass to introduce them to the Time2Focus mobile app. Initial contacts were via email, and patients were asked to complete a baseline survey before they were sent instructions on how to download the app. Participants were instructed on the use of the app, and they were provided technical support as needed while they went through the 12 levels. The treatment group included all participants who successfully downloaded the app (n=100) and were eligible to begin interacting with it (regardless of completion of any level). Participants who consented to participate but did not download the app or complete any modules (n=101) comprised the control group for this analysis. Defining the control group in this way capitalized on the fact that both groups would have a similar willingness and ability to participate as the intervention group. Participant attrition that led to the final numbers in each cohort are discussed in further detail below.

### Intervention

The goal of the Time2Focus app was to improve HbA1c, and patient confidence and skills in effectively managing diabetes by providing actionable, real-world guidance for diet, exercise, and glucose monitoring. The app was empirically based and incorporated components from evidence-based behavioral techniques, including goal-setting, problem-solving, feedback and reinforcement, and motivational interviewing. The app also leverages gamification principles to drive patients’ self-management behaviors, including incentivizing participants’ progress through positive feedback (positive messages when successfully completing a task), gradually adding complexity, and rewarding success (points earned for each module completed). Points are assigned for each task completed and accumulate over time. The total number of points through the course of use of the app served as a reminder to the participant regarding how far they progressed. There were also multiple challenges built into the app in the form of games.

The experience guided patients through progressive skill-building activities related to real-world situations. Unlike current mobile apps for type 2 diabetes, Time2Focus goes beyond simple tracking and patient education. The app was designed with the aim of improving patients’ confidence (ie, self-efficacy) in their ability to carry out tasks, build problem-solving skills, and enhance goal-setting. Points earned and feedback given served to incentivize the key skills known to help support patient skill-building.

Time2Focus consists of 12 levels designed to enhance patients’ skills and motivation with respect to several aspects of managing type 2 diabetes (eg, healthy eating, physical activity, self-monitoring blood glucose) (see Table 1).

The Time2Focus app was designed to have participants complete one level each week for a total of 12 weeks. Each level, on average, was designed to take about 1 hour to complete. Participants could start and stop each level as they wished, thereby personalizing their experience in using the app. Personalization was a central component in the app design because offering choice in the way one interacts with an intervention has been shown to promote autonomy, which in turn enhances intrinsic motivation for use of digital health interventions and engagement with behavior change [6]. Each level contained an assortment of articles, videos, challenges, and tracking tools. Once a participant completed the necessary requirements to finish each level, they received a text and email message to let them know when the next level would unlock and be available for them to start.

As part of the framework, Time2Focus encourages participants to build their skills through challenges. Participants had to attain a minimum score of 7 out of 10 on all challenges to progress to the next level. Participants who scored less than 70% could retake the challenges until they received a passing score.

Once the participants finished all 12 levels, they were sent a follow-up survey to complete. The follow-up survey included the same questions as found in the baseline survey with the addition of questions to assess the Time2Focus program. After completion of the follow-up survey, the participants received a congratulatory email, a US $25 gift card for compensation for completing each survey (totaling up to US $50), and entry to a raffle to win one of five iPad Mini tablets (ie, a participant who completed all 12 levels earned 12 entries into the raffle). Each participant was aware at the time they signed consent that they would receive a gift card and could possibly win an iPad Mini. The final gift was designed to be an incentive for completing the study.

**Table 1:** Summary of level learning objectives.

Level	Learning objectives
Level 1: Time2Focus Basics	Explain type 2 diabetes and the roles of insulin and blood glucose in the body
Level 2: Focus on Carbs	Distinguish carbohydrates from other types of food
Level 3: Focus on Physical Activity	Explain the effect of physical activity on blood glucose in type 2 diabetes
Level 4: Focus on Monitoring	Learn how to self-monitor and track blood glucose
Level 5: Mastery Challenges 1	Assess knowledge and comprehension of key concepts, including type 2 diabetes, insulin, and blood glucose
Level 6: Focus on Carb Planning	Identify targets for total carbohydrates for meals and snacks (based on carbohydrate counting)
Level 7: Focus on Making Choices	Identify protein foods, including carbohydrate proteins, and serving sizes
Level 8: Focus on Day-to-Day Decisions	Explain strategies for planning ahead for carbohydrate decisions, physical activity, and self-monitoring
Level 9: Making Decisions Away From Home	Identify strategies for planning and making decisions away from home
Level 10: Mastery Challenges 2	Assess knowledge and comprehension of key information and concepts, including nutrition, physical activity, and self-monitoring
Level 11: Keep Your Momentum Going	Establish habits, encourage decision-making, and facilitate goal-setting in managing type 2 diabetes
Level 12: Focus on the Future	Provide motivation and encouragement to patients that type 2 diabetes can be managed

### Demographic Variables

Demographic and other baseline variables collected included race, sex, age, weight, BMI, and hypertension as recorded in the electronic health record (EHR).

### Engagement

The degree of engagement was defined a priori based on the number of levels of the app that the participant completed. Levels of engagement were broken down into the following groups: participants who completed 0 levels, 1 to 4 levels, 5 to 11 levels, and 12 levels. Level 5 was the first mastery level to test what the participants had learned and to demonstrate their ability to apply these new skills. This level consisted of challenges only, and did not include articles or videos. Completing levels 5 and above indicated a markedly increased level of engagement. Thus, we a priori dichotomized engagement as low (downloading the app and completing anywhere from 0 to 4 levels) or high (completing level 5 and above).

### Primary Outcome

The primary outcome was a change in HbA1c after participants concluded their use of the app. HbA1c data were abstracted from the EHR, utilizing naturally occurring measurements obtained in the normal course of clinical care.

### Secondary Outcomes: Change in Self-Reported Health Behaviors and Perceptions of the App

Each study participant was required to complete the baseline survey before downloading the Time2Focus app and starting the program. The survey took about 10 minutes to complete, which gathered information regarding the following secondary outcomes: medication adherence, self-care activities, self-report of physical activity, diabetes self-efficacy, illness perceptions, diabetes distress scale, and users’ engagement with and rating of the app.

The Voils scale (scores from 1 to 5) was used to measure medication adherence [7]; a higher score indicates that an individual is less adherent to medications. The Stanford scale was used to measure diabetes self-efficacy [8]; a higher score (scale from 1 to 10) denotes that an individual possesses more self-efficacy to manage their diabetes. Illness perceptions were measured using the Brief Illness Perception Questionnaire [9]; a higher score (scale from 0 to 10) denotes more severe perceptions about one’s illness and has been shown to be associated with lowered perceptions of one’s ability to cope with illness. Diabetes distress was measured using 4 questions from the Diabetes Distress Scale (scale from 0 to 5) [10]; a higher score communicates that an individual has more diabetes-related stress. The baseline survey also included questions about self-care activities (eg, how often they engaged in self-care per week, and how often on average they checked their blood sugar) and self-reported physical activity (a higher score means more physical activity reported).

### Analysis

Independent-sample *t* tests and χ^2^ tests were used to examine differences in baseline characteristics for continuous and dichotomous variables, respectively, across treatment and control groups as well as between participants with low and high levels of engagement. To assess change over time in HbA1c and self-reported health behaviors, regression analyses were used, controlling for baseline values of each outcome of interest. To facilitate interpretation, the dichotomous treatment variable in all regression analyses was dummy-coded such that “0” represented the control group and “1” represented the treatment group. In all regression analyses, including those with interaction terms to test the moderation of effects, all dichotomous and categorical covariates (with the exception of treatment) were coded effects such that the regression weights represent a comparison of each group with the average across all groups. For different analyses, mean clinical values, changes in mean clinical values, standardized β weights, or Cohen *d* effect size, estimates are presented to maximize interpretation. All analyses were performed in R version 3.5.3 (R Foundation for Statistical Computing, Vienna, Austria).

## Results

### Study Population, Recruitment, and Enrollment

Seventy-eight primary care providers representing 23 different adult continuity practices gave permission for their patients to be contacted about this study. Of the 1355 potentially eligible patients screened, 780 were ineligible, 169 declined, 205 could not be reached, and 201 were consented. Among the 201 consented participants, 143 (71.1%) completed the baseline survey and 100 (49.8%) downloaded the Time2Focus app, who were eligible to start the 12-week Time2Focus program.

### Baseline Demographics and Survey Results

The baseline characteristics of the participants are summarized in Table 2. The participants on average were 57.3 years old, 54.7% (110/201) were white, and there was an approximately equal sex ratio. They had a mean BMI of 35.8 and HbA1c of 9.02%. Among the 201 participants, over half had hypertension and approximately 10% had hyperlipidemia. These characteristics were also compared across app downloaders (100/201, 49.8%) and nondownloaders (101/201, 50.2%). Nonwhite participants were significantly less likely to download the app than white participants (χ^2^=4.25, *P*=.04); however, no other baseline characteristics were significantly different across app downloaders and nondownloaders (Table 2).

**Table 2:** Baseline characteristics across levels of engagement.

Baseline characteristics	Consented (N=201)	App nondownloaders (n=101)	App downloaders (n=100)	Completed <level 5 (n=42)	Completed ≥level 5 (n=58)
Age (years), mean (SD)	57.30 (10.92)	58.52 (10.75)	56.06 (11.00)	57.33 (11.58)	55.14 (10.57)
Nonwhite, n (%)	91 (45.3)	53 (52.5)	38 (38.0)	15 (35.7)	23 (39.7)
Female, n (%)	100 (49.8)	51 (50.5)	49 (49.0)	20 (47.6)	29 (50.0)
Weight (kg), mean (SD)	104.5 (26.49)	99.40 (27.58)	106.63 (25.84)	103.82 (26.08)	108.66 (25.70)
BMI (kg/m^2^), mean (SD)	35.8 (7.62)	34.82 (7.89)	36.25 (7.50)	35.11 (7.77)	37.09 (7.26)
Hypertension, n (%)	115 (57.2)	58 (57.4)	57 (57.0)	26 (61.95)	31 (53.4)
Hyperlipidemia, n (%)	22 (10.9)	9 (8.9)	13 (13.0)	6 (14.3)	7 (12.1)
Hemoglobin A1c (%), mean (SD)	9.02 (1.17)	9.04 (1.17)	9.00 (1.17)	9.05 (1.22)	8.96 (1.15)

Baseline survey results for the 143 participants (71.1% of consented participants) who filled out the survey are shown in Table 3. The majority of patients were taking medications, and they rated their nonadherence as minimal, reported a moderate amount of physical activity, rated their diabetes self-efficacy as moderate, and rated their diabetes distress as somewhat elevated. These baseline survey results were also compared across app downloaders and nondownloaders. Participants who did not download the app had significantly higher diabetes self-efficacy than participants who downloaded the app (t_199_=2.62, *P*=.009). There were no other differences regarding baseline survey results between app downloaders and nondownloaders (Table 3).

**Table 3:** Survey results across levels of engagement.

Survey question	All survey respondents (N=143)	App nondownloaders (n=43)	App downloaders (n=100)	Completed <level 5 (n=42)	Completed ≥level 5 (n=58)
Currently taking medication, n (%)	140 (97.9)	43 (100)	97 (97)	41 (97.6)	56 (96.6)
Extent of medication nonadherence, mean (SD)	1.68 (0.75)	1.60 (0.58)	1.17 (0.82)	1.63 (0.89)	1.78 (0.76)
Self-report physical activity measures, mean (SD)	3.14 (0.69)	2.85 (1.17)	2.55 (1.12)	2.66 (1.03)	2.48 (1.19)
Diabetes self-efficacy scale, mean (SD)	6.72 (1.76)	7.17 (1.71)	6.53 (1.75)	6.73 (1.93)	6.38 (1.61)
Brief Illness Perceptions Questionnaire, mean (SD)	5.28 (1.30)	5.13 (1.38)	5.40 (1.14)	5.13 (1.16)	5.59 (1.11)
Diabetes Distress Scale, mean (SD)	2.09 (1.20)	2.00 (1.27)	2.13 (1.17)	2.14 (1.21)	2.13 (1.15)
Doctor-recommended home glucose test, n (%)	142 (99.3)	43 (100)	99 (99)	41 (97.6)	58 (100)
Self-care: days per week blood glucose checked, mean (SD)	4.13 (2.62)	3.84 (2.64)	4.26 (2.62)	4.12 (2.79)	4.36 (2.51)

### Engagement

Overall, 48 of the 201 participants (23.9%) who consented to participate completed all levels and the follow-up survey, and 100 (49.8%) of those who consented to participate downloaded the app. Of the 100 participants who downloaded the app, 16 (16.0%) completed 0 levels, 26 (26.0%) completed 1 to 4 levels, 10 (10.0%) completed 5 to 11 levels, and 48 (48.0%) completed all 12 levels of the app and the follow-up survey.

There were no significant or descriptively meaningful differences among participants who completed 0 to 4 levels of the app. Similarly, there were no significant or descriptively meaningful differences among participants who completed 5 to 12 levels of the app (see Table 3). Because of the a priori distinction that the completion of level 5 or above indicates a markedly increased level of engagement, all subsequent analyses related to engagement examined engagement as a dichotomous variable. As shown in Table 2, baseline characteristics were not significantly different across high engagers (completed level 5 or higher) and low engagers (completed 0 to 4 levels). At baseline, high engagers (as compared to low engagers) had more severe perceptions of illness on their Brief Illness Perceptions Questionnaire (t_92_=2.01, *P*=.047).

### Primary Outcome: Change in HbA1c

**Figure 1:**
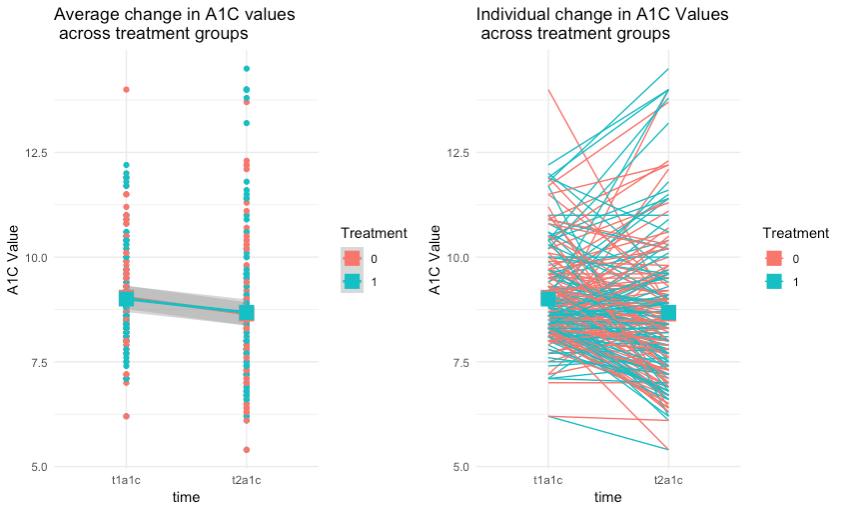
Average individual change in hemoglobin A1c (A1C) by group.

Figure 1 illustrates the change in HbA1c of all participants, both as an average across app downloaders and nondownloaders (left panel) and as individual trajectories of HbA1c change (right panel).

When adjusting for baseline levels of HbA1c, HbA1c did not change more significantly for the treatment group (–0.32) than for the control group (–0.39; β=.06, *P*=.78). However, higher baseline levels of HbA1c were significantly related to greater decreases in HbA1c (β=–.33, *P*=.001). This significant association is common and is usually assumed to be evidence of regression toward the mean. The null effect of treatment also held when examining a subgroup of participants with a baseline HbA1c greater than 8% (β=.15, *P*=.53). Subgroup analyses of HbA1c and engagement were also conducted. The interaction between baseline HbA1c and the number of levels engaged with in the app (β=.02, *P*=.45) was not associated with change in HbA1c. Thus, when controlling for baseline HbA1c, high versus low engagers did not experience significantly different changes in HbA1c. Additionally, those completing one or more levels of the app (mean pre/post-HbA1c change of –0.41%) did not have significantly different changes in HbA1c compared with those who downloaded the app but completed 0 levels (mean pre/post-HbA1c change of –0.32%; *P*=.73).

### Secondary Outcomes: Change in Self-Reported Health Behaviors

Because follow-up measures of self-reported health behaviors were only based on participants who completed the app levels, comparisons in changes in self-reported health behaviors across treatment and control participants could not be performed. However, changes in self-reported health behaviors for the 48 participants completing all levels of the app were examined. Diabetes self-efficacy showed a large and significant increase during app usage (mean change 1.28, *P*<.001, *d*=.83). Severity of illness perceptions showed a small but significant decrease during app usage (mean change –0.51, *P*=.004, *d*=.43). Diabetes distress showed a small but significant decrease during app usage (mean change –0.45, *P*=.006, *d*=0.41). Self-reported medication nonadherence (mean change –0.18, *P*=.11) and physical activity (mean change 0.24, *P*=.14) did not significantly change during app usage. These changes in self-reported health behaviors were consistent across gender (all *P*>.19), race (all *P*>.30), and BMI (all *P*>.27). Increased age was marginally associated with greater improvements in diabetes self-efficacy (β=.03, *P*=.05), and positive hypertension/hyperlipidemia status was associated with greater increases in physical activity (β=.75, *P*=.006).

### User Experience/Perceptions of the Program

Those who completed all levels of the app (24% of all consented participants, 48% of app downloaders) rated it highly and would recommend it to others (net promoter score=62.5). Overall, participants who completed the app reported being highly satisfied (mean 4.56, SD 0.76; 1 to 5 scale); that the app has high relevance to their daily lives (mean 4.52, SD 0.68; 1 to 5 scale); that the app helped them better manage their type 2 diabetes (mean 4.38, SD 0.81; 1 to 5 scale); and strongly endorsed that they would recommend this app to colleagues, friends, or family with diabetes (mean 9.06, SD 1.46; 1 to 10 scale). Features of the app that were endorsed as among the most helpful by app completers were the healthy eating articles (n=42, 89%), food comparison tool (n=36, 77%), challenges (n=32, 68%), physical activity articles (n=31, 66%), and blood glucose monitoring articles (n=26, 55%).

## Discussion

### Principal Results

The Time2Focus mobile app is uniquely designed to assist users in building skills that are needed to reach diabetes self-management goals. Our results demonstrate that participants who used the Time2Focus mobile app showed an improvement in diabetes self-efficacy, which is an essential skill needed in the care and management of diabetes. Users also experienced a significant decrease in severity of illness perceptions. This finding is critically important, since more severe illness perceptions are associated with a stronger cognitive and emotional response to illness, which can negatively influence the perceived ability to cope with the illness [11]. In addition, more severe illness perceptions have also been associated with poorer glycemic control as measured by HbA1c [12].

A decrease in HbA1c in both the intervention and control groups was observed over the 12-week follow-up period. However, there was no difference in change in HbA1c between the intervention and control groups. Importantly, users reported high satisfaction with using the app, found the app to have high relevance to their daily lives, thought the app helped them better manage their diabetes, and said that they would recommend the app to others.

### Comparison With Prior Work

Our results mirror many other studies designed to test the effect of technology-based educational apps on HbA1c. For example, a systematic review evaluating mobile apps designed to deliver SMS demonstrated that of 11 studies selected for review, only 45.4% of the studies observed an HbA1c reduction in both the intervention and control groups [5]. Another systematic review that focused on digital health technology and mobile devices for patients with diabetes reviewed studies that were specifically focused on SMS and education for patients with diabetes [13]. Results with respect to change in HbA1c were mixed. The most efficacious studies included personal coaching or personalized messaging, in-person visits, and website components. In contrast, a systematic review performed by Greenwood et al [14] revealed that the majority (18/25) of the 25 review articles and meta-analyses included reported a significant reduction in HbA1c ranging from 0.1% to 0.8%. Communication, education, and feedback are consistently noted to be key design elements for interventions aiming to reduce HbA1c through delivery of diabetes self-management education and support [14,15].

One example of an efficacious multicomponent app is the mySugr mobile app, which was designed to support patients in healthy eating, being active, monitoring and taking medications, risk reduction, problem solving, and healthy coping skills. The features of this app include wireless blood glucose data upload, recording of insulin use and exercise, on-demand direct access to a certified diabetes educator, and algorithms for pattern detection and assistance with day-to-day diabetes management. Results showed that when participants used the mySugr “Bundle” (app plus unlimited test strips and certified diabetes educator–led coaching), there was significant improvement in mean blood glucose (–10.4%), tests in range (+8.5%), and estimated HbA1c (–0.4%) [15].

The lack of difference in HbA1c in this pilot study may be related to our small cohort size as well as our pragmatic approach to HbA1c data collection. Indeed, mobile health technologies that are efficacious when implemented in clinical trials often are not effective when implemented in the field [15,16]. In addition, we chose to utilize clinically derived data because we wanted to test the app and its effects in a real-world environment. It is possible that HbA1c collected by the study team at specific time intervals could have led to a difference in HbA1c in the intervention vs control group.

In addition, differences in socioeconomic status, technological literacy, and low health literacy are known barriers to enrollment and engagement in technology for individuals with diabetes [17,18]. Therefore, it is possible that the lack of difference in HbA1c was secondary to differences in our intervention and control groups with respect to any of the aforementioned characteristics. Lastly, the effects of SMS education can be delayed, and the time frame during which one sees the greatest impact from education and skill-building can be patient-dependent. Indeed, most mobile health trials that have shown effective HbA1c lowering were designed for follow up over an average of 6-12 months [5,14]. Therefore, it is possible that assessing HbA1c after a longer time interval (ie, 6 months) could have led to a different result.

Forty-eight participants (24% of consented participants, 48% of app downloaders) completed all levels of the app, and satisfaction with the app among all users was high. This level of engagement is on par with prior pragmatic trials that tested similar apps, and is particularly high considering that we did not utilize in-person visits or personal coaching components. It is important to note that the number of levels engaged in the app was also not associated with changes in HbA1c (β=–.01, *P*=.76).

Current published data demonstrate low levels of adoption of mobile apps that are designed to assist patients in the management of their diabetes [19]. Further, when individuals do download mobile apps, engagement rates decline over time and attrition rates are often high [19]. Unlike the Time2Focus app, one reason for lack of engagement in other apps is a lack of satisfaction with the design of the app. Our higher level of satisfaction with the app is likely a reflection of the design, which sought to build in real-world situations, allow users to move at their own pace, and is focused on skill-building and improving confidence. The design of the app is consistent with the guiding principles described in the person-based approach to intervention development. These guiding principles describe intervention features that improve acceptability and engagement in an intervention, including promoting user autonomy, competence, and positive experience [6]. Participants also noted they would recommend the app because it was “informative and educational,” “keeps up motivation levels,” and “holds me accountable.”

### Strengths

Managing diabetes is time-consuming as well as emotionally and financially burdensome. Having a mobile app option to support diabetes management and education, reduce the severity of illness perceptions, and improve diabetes self-efficacy is a step forward in providing multiple tools to help patients with diabetes achieve their SMS and glycemic goals. The strengths of our pilot study include: (1) the intervention design, which is based on behavior-change theory and focuses on increasing self-efficacy and problem-solving skills; (2) the pragmatic implementation of the intervention and collection of data; and (3) the limited resources needed to implement the intervention.

### Limitations

Some limitations of our study are mainly due to the naturalistic setting in which it was conducted, and include a small cohort, the use of naturally occurring HbA1c as the primary outcome, the short duration of the study, and lack of randomization. Other limitations of our study may be addressed in future studies even within naturalistic settings. These limitations include the inherent tying of incentives to intervention completion, and the completion of follow-up surveys in only the 48 participants (23.8%) who completed all levels of the app.

With regard to only administering follow-up surveys to participants who completed the app, it should be noted that the majority of our inferences regarding changes in self-efficacy and other self-reported measures can only be applied to those participants and not to those that dropped out early. Thus, this pilot study largely presents evidence on the amount of engagement across all participants, but changes only within participants willing to engage throughout the app. Although resource constraints influenced our ability to collect additional follow-up data from participants who dropped out, there would be tremendous utility in capturing additional feedback on why some participants dropped out. In this regard, qualitative interview methods may have been instrumental in capturing rich and valuable data on why some individuals disengaged without the need to fully survey all participants who disengaged, which may be prohibitive.

### Conclusions

Participants who used the Time2Focus mobile app showed an improvement in diabetes self-efficacy and a decrease in severity of illness perceptions. The decrease in HbA1c observed in app users relative to nonusers during this limited pilot study was not statistically significant. However, uptake and application of lessons learned from SMS may be delayed. Future research is needed to address how to increase engagement in SMS and to investigate if follow up over a longer period would result in a significant change in clinical outcomes such HbA1c.

## Abbreviations

EHR: electronic health record
HbA1c: hemoglobin A1c
SMS: self-management support
